# Pattern of Mandibular Third Molar Impaction in Nonsyndromic 17760 Patients: A Retrospective Study among Saudi Population in Central Region, Saudi Arabia

**DOI:** 10.1155/2021/1880750

**Published:** 2021-08-26

**Authors:** Mahmud Uz Zaman, Naif Salem Almutairi, Mohammed Abdulrahman Alnashwan, Shabab Mohammed Albogami, Nawaf Masad Alkhammash, Mohammad Khursheed Alam

**Affiliations:** ^1^Department of Oral Medicine and Diagnostic Sciences, College of Dentistry, King Saud University, Riyadh, Saudi Arabia; ^2^Dental University Hospital, College of Dentistry, King Saud University, Riyadh, Saudi Arabia; ^3^Department of Preventive Dental Science, College of Dentistry, Jouf University, Sakaka, Saudi Arabia

## Abstract

The objective of this study was to study the incidence of type of impaction of mandibular third molars based on the classifications of Pell and Gregory and Winter, which included angulation of the tooth and level of the occlusal surface of the third molar with respect to the second molar, respectively, in a sample of Saudi population in central region. In this retrospective study, orthopantomograms (OPGs) of 17760 patients were examined, who were reported by the Dental University Hospital (DUH) at King Saud University, Riyadh, Saudi Arabia, between the years 2016 and 2020. Out of 17760 radiographs, 2187 (12.31%) patients presented with at least one impacted third molar. Out of which, 1337 (7.52%) patients had bilateral impaction and 850 (4.78%) patients had unilateral impaction (*p* < 0.001). No gender predominance was noted in the impaction status (*p* > 0.05). In bilateral impaction, 671 were male (50.2%) and 666 were female (49.8%). Among unilateral impaction, 394 (46.4%) were male and 456 (53.6%) were female. Mesioangular angulation was the most common pattern of impaction (65%) followed by vertical angulation in both bilateral and unilateral impactions. Level A impaction was found to be highest in both bilateral and unilateral impactions which are 48.02% and 54.0%, respectively (*p* < 0.05). Our study highlights mesioangular impaction and level “A” as the most frequently encountered angulation and level of impaction in impacted teeth. This study result provides us useful data regarding the radiographic status of mandibular third molars in the population of Saudi Arabia.

## 1. Introduction

Tooth impaction is an abnormal condition, which is characterized by failure of eruption of the tooth in the oral cavity within a standard time. The reason for impaction may be due to lack of space in the arch or physical barriers like obstruction by another tooth in the eruptive pathway of the tooth or developed in an abnormal position [1–4]. British Association of Oral and Maxillofacial Surgeons and the Royal College of Surgeons of England Faculty of Dental Surgery published a guideline which included definitions of an unerupted tooth which is lying in the jaws, partially or completely covered by the bone or soft tissue interfered with by other teeth [3]. Maxillary and mandibular third molars, maxillary cuspids, and maxillary central incisors are the most frequently impacted teeth [3]. The lack of space between the teeth along with the tendency of third molars to erupt late in the order of tooth eruption explains the fact that the third molars are the most frequently ‘impacted teeth.' [5] Third molars are the most often congenitally missing teeth but 90% of the population has impacted teeth among them; 33% have at least one impacted third molar [6]. Any tooth can be impacted in the oral cavity, but out of all impaction, the third molar accounts for 98% [1, 2]. They are directly or indirectly associated with numerous disorders in the mouth, jaw, and facial regions such as caries, pericoronitis, cystic lesions, periodontitis, neoplasms, or root resorption [1, 7–9]. Therefore, the extraction of third molars is one of the most common surgical procedures for oral and maxillofacial surgeons [10].

To date, several impaction classification methods have been used, in which authors tried to describe the impaction based on the angulations of the third molars [11], the level of impaction, and the relationship to the anterior border of the ramus of the mandible [12], and Winter's [11] and Pell and Gregory [12] classifications are most commonly used to classify impacted mandibular third molars. In Winter's classification, the angulation of impaction of the mandibular third molar is determined by the angle formed between the intersected longitudinal axes of the mandibular second and third molars [11]. Level (depth) of impaction can be classified using the Pell and Gregory classification system, where the impacted teeth are assessed according to their relationship to the occlusal surface of the adjacent second molar [13].

In Saudi Arabia, the pattern of third molar impaction has been assessed in several other regions [14–16], excluding two studies by Haidar and Shalhoub [17] and Alfadil and Almajed [18] which were conducted in the central region 35 years ago and two years ago, respectively. Haidar and Shalhoub [17]evaluated 1000 panoramic radiographs and reported 32.3% for third molar impaction incidence without any sex predilection. But they only determined the angulation of impaction; level of impaction was not assessed at that time. The aim of the out study was to determine the angulation, level, and position of the impacted lower third molars among Saudi population in Central Region, Saudi Arabia. This identification of the impaction pattern of teeth would help to predict whether the impacted teeth will erupt or will remain impacted; therefore, it might help the oral and maxillofacial surgeons to take a clinical decision regarding the best timing of their extraction or any other treatment plan according to the condition of impaction.

## 2. Materials and Methods

This study was approved by the Institutional Review Board (IRB) of King Saud University (KSU). A total of 17760 orthopantomographs (OPGs) of Saudi patients were retrieved; all OPGs were taken in the Dental University Hospital (DUH) at KSU, Riyadh, Saudi Arabia, between the years 2016 and 2020.

The inclusion criteria were as follows:

- All lower first and second molars were appropriately aligned in the dental arch
- Impacted lower third molars with completed root formation with clear root apecies on the OPGs
- Patients should be healthy with no systemic diseases or disorders
- No history of any lower third molar extraction

A total of 15573 OPGs were excluded for the following reasons:

- Incomplete patient record or poor image quality
- Aged below 20
- Caries or periapical pathosis or any other abnormality in the lower third molars
- Previous history of orthodontic treatment
- Absence of second molar or mesially drifted lower third molar due to loss of second or first molars
- Craniofacial anomalies or syndromes
- Any history of trauma to the jaw

All OPGs were taken by PLANMECA ProMax Digital S3 machine, and measurements were performed using Planmeca Romexis® 5.2.0.R imaging software (Planmeca, Helsinki, Finland). Images were viewed on a 30.4-inch TFT AM Color LCD Dual Domain IPS medical display Coronis Fusion MDCC-6130 (Barco, Belgium) at a resolution of 3280 × 2048. All measurements and analyses were performed by four 5^th^ year dental students who were trained by one experienced oral and maxillofacial radiologist.

The following definitions were used for assessment of angular position and level of eruption.

### 2.1. Angular Position

**Figure 1:**
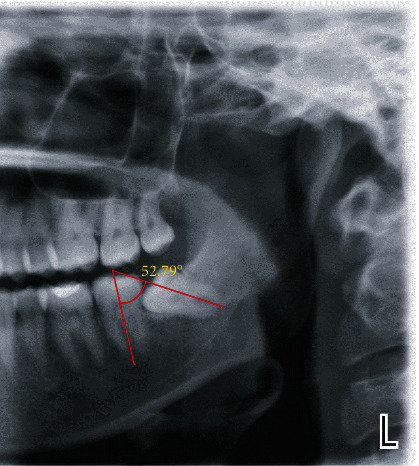
Measurement of angulation of third molar on panoramic radiograph.

The angulation of impacted lower third molars was assessed by drawing lines in the panoramic radiographs in the Planmeca Romexis® imaging software according to Winter's classification [11]. Through long axis of the second and third molars, two lines were drawn (Figure 1). The angle formed by the intersection of those lines was measured automatically by Planmeca Romexis® 5.2.0.R imaging software. The following angular classification was used to avoid errors arising from visual impression:

- Vertical impaction: 10° to -10°
- Mesioangular impaction: 11° to 79°
- Horizontal impaction: 80° to 100°
- Distoangular impaction: -11° to -79°
- Other (inverted, buccolingual): 111° to -80°

### 2.2. Level of Eruption

The level of eruption was documented according to the relationship between the occlusal surface of the third molar and the cementoenamel junction of the adjacent second molar mentioned in the Pell and Gregory classification [12] and was as follows:

- Position A: the highest position of the impacted third molar was on the same level or above the occlusal plane of the adjacent second molar
- Position B: the highest position of the impacted third molar was located below the occlusal plane but above the cervical line of the adjacent second molar
- Position C: the highest position of the impacted third molar was below the cervical line of the adjacent second molar

### 2.3. Statistical Analysis

Data analysis was completed using SPSS 23.0 software (SPSS Inc., Chicago, IL). The prevalence of impacted third molars in relation to age, gender, type of impaction, and level of impaction was evaluated and expressed as frequency and percentage. Chi square test and *t*-test were used to compare statistical differences. A *p* value less than 0.05 was considered statistically significant with a confidence interval set at 95%. Intra- and interexaminer reproducibility was assessed by retracing 100 OPGs with a one-month interval. For reliability, Cronbach's alpha interpretation of internal consistency was used (Table 1).

## 3. Results

Total number of OPGs retrieved for this study was 17760. Out of those, 2187 patients had impacted lower third molars. Out of which, 1337 (7.52%) subjects had bilateral impaction. And 850 (4.78%) subjects had unilateral impaction. This prevalence of bilateral and unilateral impactions among study population was statistically highly significant (*χ*^2^ = 216.89, *p* < 0.001 HS). The overall prevalence of impaction among study subjects was 12.31% (Table 2).

Out of 850 unilateral impactions, 416 (48.94%) were lower left third molar (#38) and 434 (51.06%) were lower right third molar (#48). There was no statistical significant (*p* > 0.05) difference between the right and left sides (*χ*^2^ = 0.762, *p* = 0.410 NS) (Table 3).

In bilateral impaction, 671 were male (50.2%) and 666 were female (49.8%). Among unilateral impaction, 394 (46.4%) were male and 456 (53.6%) were females. This difference was statistically nonsignificant (*p* > 0.05) according to gender (*χ*^2^ = 3.058, *p* = 0.087 NS) (Table 4). In unilateral impaction, 191 were male (45.91%) and 225 were female (54.09%) in #38. In #48, 203 (46.77%) were male and 231 (53.22%) were female. This difference was also statistically nonsignificant (*p* > 0.05) according to gender (*χ*^2^ = 0.063, *p* = 0.8370 NS) (Table 5).

The mean age of study subjects in bilateral impaction was 26.86 ± 7.94, and in unilateral impaction, it was 31.16 ± 10.19 (Table 6). The mean age of study subjects in #38 was 30.45 ± 9.44 (*t* = 0.066, *p* = 0.948 NS), and in #48, it was 31.85 ± 10.82 (*t* = 0.192, *p* = 0.848 NS) (Table 7). The mean age according to gender in bilateral (*t* = 0.112, *p* = 0.911 NS) and unilateral (*t* = 0.086, *p* = 0.931 NS) impactions was found to be statistically nonsignificant (*p* > 0.05) (Table 6).

The prevalence of angular position is tabulated in (Tables 8 and 9). In both bilateral and unilateral impactions, mesioangular type of impaction was found to be more, accounting 41.07% and 39.18%, respectively. Next to mesioangular impaction, it was the vertical type of impaction accounting 39.11% in bilateral impaction and 38.82% in unilateral impaction, followed by distoangular 15.63% and 17.53%, horizontal 3.82% and 3.88%, and others 0.37% and 0.59%, respectively, in descending order. Interestingly, #38 vertical type of impaction was found to be little more, accounting 38.46%, followed by mesioangular 37.74%, distoangular 19%, and horizontal 4.33%, and other angulation was present in less than 0.47% of cases. On the other hand, in #48 mesioangular type of impaction was found to be more, accounting 40.55%, followed by vertical 39.17%, distoangular 16.13%, and horizontal 3.46%, and other angulation was present in less than 0.69% of cases in descending order. The least type of angular impaction excluding other type was horizontal impaction in both bilateral and unilateral impactions. These differences in angulation according to unilateral or bilateral impaction (*χ*^2^ = 2.140, *p* = 0.710 NS) and also between tooth numbers within unilateral impactions (*χ*^2^ = 2.023, *p* = 0.731 NS) were found to be statistically nonsignificant (*p* > 0.05).

The level of eruption evaluation is shown in (Tables 10 and 11). In bilateral and unilateral impactions, level A type of impaction was found to be more, accounting 48.02% and 54.0%, respectively. Next to level A impaction, it was level B type of impaction accounting 32.98% in bilateral impaction and 27.05% in unilateral impaction, followed by level C 19% and 18.95% for bilateral and unilateral impactions, respectively. In #38 and #48 also, level A was the most common type of impaction accounting for 54.08% and 53.45%, respectively, followed by level B accounting for 26.93% and 27.19%. The least level of impaction was level C, accounting for 18.99% in #38 and 19.36% in #48. The differences in level of angulation according to unilateral or bilateral impaction (*χ*^2^ = 9.641, *p* = 0.008 S) were found to be statistically significant (*p* < 0.05), whereas between tooth numbers within unilateral impaction (*χ*^2^ = 0.036, *p* = 0.982 NS), the difference was found to be statistically nonsignificant (*p* > 0.05). Based on angulation of impaction and level of impaction, the intra- and interexaminer reliability was found to be excellent >0.9 (Tables 12–14).

## 4. Discussion

The mandibular third molars are the most frequently impacted teeth and surgical extraction; these molars become one of the most common dentoalveolar surgeries [10, 19]. The etiology of impaction depends upon several factors [20]. Byahatti and Ingafou [21] suggested various reasons like physical disruption of the dental lamina, space limitation, and an inherent defect of the dental lamina, or failure of induction of the underlying mesenchyme. Third molar impaction may be associated with periodontal disease, dental caries, odontogenic cyst and tumors, pain of unexplained origin, jaw fracture, and resorption of root of the adjacent tooth [22]. Generally, the risks of surgical third molar removal are very little, such as pain, bleeding, infection, swelling, and dry socket. But sometimes, serious complications may occur, such as injury to the temporomandibular joint, trismus, or permanent paresthesia. It is necessary to assess the prognosis of impacted third molars and its eruption to avoid unnecessary complications associated with these retained teeth. Because of the increasing incidence of unerupted third molars, it is recommended to use intraoral periapical radiographs and cone-beam computerized tomography for assessing the proximity of impacted mandibular third molar roots to the inferior alveolar canal. The chances of damaging the inferior alveolar nerve during surgery may reduce.

This is the third study to evaluate incidence of impacted third mandibular molars in the population of the Central Region of Saudi Arabia. Previous two studies were conducted 35 years and two years ago, respectively [17, 18]. The number of patient's radiographs in our study was equivalent or more to the number of radiographs used in many other national and international studies [14–18, 23–38], and inclusion and exclusion criteria of patients were almost similar like other studies which allowed us to compare our results with such studies.

Studies showing the bilateral and unilateral occurrences of impacted third molars are very rare. Dachi and Howells [35] found that unilateral and bilateral impactions of third molars occurred with almost equal frequency. In our study, we found that the frequency of bilateral impaction is a little higher than unilateral impaction (*χ*^2^ = 216.89, *p* < 0.001 HS). Quek et al. [23] also mentioned that bilateral occurrence of third molars was more common than unilateral impactions in their study.

The mean age of our cases was 26.86 years in bilateral impaction and 31.16 years in unilateral impaction, which is almost similar to the average age as reported for the eruption of mandibular third molar [36, 39]. Schersten et al. [40] suggested that 20 to 25 years is the most suitable age for studying the frequency of the mandibular third molar and its impaction.

Like most of the other studies, we found no statistically significant (*p* > 0.05) gender distribution in the prevalence of mandibular third molar impaction [24, 25, 34–38]. But few others found higher frequency of third molar impactions in female than male [23, 26, 27, 29, 41, 42]. The reason for female predominance was described by Hellman [39]. He stated that higher frequency reported in females is due to the consequence of difference between the growth of male and female. Females usually stop growing when the third molars just begin to erupt, whereas in males, the growth of the jaws continues during the time of eruption of the third molars, creating more space for third molar eruption.

The prevalence of patients with minimum one impacted third molar in our study was 5%, which is in disagreement with findings of other authors: Bokhari et al. 19% [16], Hassan 40% [15],Rajasuo et al. 38% [28], Sadeta et al. 38% [29], Hattab et al. 33% [30], and Eliasson et al. 30% [31]. Haider and Shalhoub [17] reported 34% and 29% prevalence of impacted third molars for males and females, respectively. Alfadil and Almajed [18] reported 30.9% patients had at least one impacted third molar. This discrepancy perhaps is due to the higher number of patients considered for our study. Also, we only focused our study for lower third molars.

Different classification systems were used across studies. So, it is difficult to compare the prevalence of the different angulations of impaction. Moreover, most studies measured angulation of impaction by visual impression alone. In our study, we found the mesioangular impaction is the most common type of impaction in both unilateral 39% and bilateral impactions 41%, respectively. Our study result shows similar results found in various other studies [14–16, 18] and international studies [11, 15, 31–34, 36]. Bokhari et al. [16] mentioned about Belfast Study Group in their article. According to them, there may be differential root growth between the mesial and distal roots, which causes the root to either remain mesially inclined or rotate to a vertical position, depending on the amount of root development. Higher prevalence of mesioangular impaction may be related to the more common underdevelopment of mesial root. But we found vertical angulation is not so far behind, unilateral 39% and bilateral impactions 39%, respectively, Alfadil and Almajed [18] also got similar results in their study. A previous study conducted in the same region by Haidar and Shalhoub [17] noted more frequency of vertical impaction 53.9% followed by mesioangular 32.7%. Hugoson and Kugelberg [26] also reported vertical impaction and mesioangular impactions of 50% and 30%, respectively. In our findings, horizontal impaction is least common 3.82% in bilateral and 3.88% in unilateral impaction, the same as Haidar and Shalhoub [17] horizontal 5.1%. Alfergani et al. [14] and Hassan [15] found horizontal impaction 23.5% and 27.5% 2nd most common type of impaction after mesioangular impaction 43.3% and 33.4%, respectively. Hassan [15]and Bokhari et al. [16] reported distoangular impaction as 16.6% and 1.4%, respectively, and is the least common in Saudi population. Alfadil and Almajed [18] found distoangular 3.1% is the least common type of angulation in this region which is in complete disagreement with our study.

For the assessment of level of impaction according to Pell and Gregory classification, the most common pattern of impacted mandibular third molars was in level B [12]. Many studies across the globe have shown contradicting results. Marwa et al. 44.7% [42], Quek et al. 80% [23], Sandhu and Kaur 39% [43], and Padhye et al. 45.8% [36], all have shown similar results of Pell and Gregory classification [12]. Previous two studies conducted in Saudi Arabia, in different regions, also showed similar results: Alfergani et al. 68% [14] and Hassan 67.7% [15]. But, in our study, it showed that the majority of the patients had level A impaction more (statistically significant *p* < 0.05), accounting for 48.02% and 54% in bilateral and unilateral impactions, respectively. This finding was found to be more common in studies of Hattab et al. 58% [30] and Gupta et al. 61.8% [34]. But Alfadil and Almajed [18] found level C is the most common type of level of impaction in this region, which is contradictory to our study result.

The results of this study would help the oral and maxillofacial surgeons of the central region of Saudi Arabia for patient's evaluation and also emphasize the need of understanding the variations of pattern of impacted lower third molar globally. A large population of young individuals may have one or more impactions. The prevalence and types of impactions may vary in different racial and ethnic groups. It is, therefore, imperative to understand the pattern of impactions in various communities and population subgroups. All these clinical and radiographic variables are important to consider prior to the surgery. They will help determine the difficulty of surgical procedure, duration of the surgery, expected postoperative complications, type of anesthesia, and selection of the preoperative or postoperative medications.

All the previous studies performed in Saudi Arabia had less number of patients except Alfadil and Almajed's report [18]. But we found few contradictory results compared with Alfadil and Almajed's study [18] results. Thus, it may be said that our study results represent the latest pattern of impacted third molars in a group of Saudi population.

## 5. Conclusion

The pattern of lower third molar impaction in the present sample was characterized by no significant difference (*p* > 0.05) in incidence of impaction between females and males. The most common angulation was the mesioangular angulation followed by vertical angulation. The most common level of impaction was level A. There was no significant difference between the right and left sides (*p* > 0.05). Future studies are required to evaluate the pattern of maxillary third molar impaction in Saudi Arabia and impacted third molars relation with surrounding vital structures.

## Figures and Tables

**Table 1:** Interexaminer reliability Cronbach's alpha interpretation of internal consistency table.

Cronbach's alpha	Internal consistency
*α* ≥ 0.9	Excellent
0.9 > *α* ≥ 0.8	Good
0.8 > *α* ≥ 0.7	Acceptable
0.7 > *α* ≥ 0.6	Questionable
0.6 > *α* ≥ 0.5	Poor
0.5 > *α*	Unacceptable

**Table 2:** Distribution of total number of patient's in bilateral and unilateral impactions. Data presented as frequency and percentage.

Total number of patients	Bilateral impaction		Unilateral impaction	
	Frequency	Percentage	Frequency	Percentage
17760	1337	7.52	850	4.78

*χ*^2^ = 216.89, *p* < 0.001 HS.

**Table 3:** Distribution of total number of patient's in unilateral impaction. Data presented as frequency and percentage.

Unilateral impaction (*n* = 850)			
Tooth number (38)		Tooth number (48)	
Frequency	Percentage	Frequency	Percentage
416	48.94	434	51.06

*χ*^2^ = 0.762, *p* = 0.410 NS.

**Table 4:** Gender distribution of total number of patient's in bilateral and unilateral impactions. Data presented as frequency and percentage.

Gender	Bilateral impaction		Unilateral impaction	
	Frequency	Percent	Frequency	Percent
Male	671	50.2	394	46.4
Female	666	49.8	456	53.6
Total	1337	100.0	850	100.0

*χ*^2^ = 3.058, *p* = 0.087 NS.

**Table 5:** Gender distribution of total number of patient's in unilateral impaction. Data presented as frequency and percentage.

Unilateral impaction (*n* = 850)				
Gender	Tooth number (38)		Tooth number (48)	
	Frequency	Percent	Frequency	Percent
Male	191	45.91	203	46.4
Female	225	54.09	231	53.6
Total	416	100.0	434	100.0

*χ*^2^ = 0.063, *p* = 0.837 NS.

**Table 6:** Mean age of patients according to type of impaction and gender distribution.

Gender	Bilateral impaction			Unilateral impaction		
	*N*	Mean age	Std. deviation	*N*	Mean age	Std. deviation
Male	671	26.85	8.24	394	31.1371	10.40
Female	666	26.90	7.57	456	31.1974	10.01
Total	1337	26.86	7.94	850	31.16	10.19

*t* = 0.112, *p* = 0.911 NS; *t* = 0.086, *p* = 0.931 NS.

**Table 7:** Mean age of patients in unilateral impaction and gender distribution.

Tooth number in unilateral impaction (*n* = 850)						
Gender	Lower left third molar (38)			Lower right third molar (48)		
	*N*	Mean age	Std. deviation	*N*	Mean age	Std. deviation
Male	191	30.49	9.80	203	31.74	10.92
Female	225	30.43	9.14	231	31.94	10.76
Total	416	30.45	9.44	434	31.85	10.82

*t* = 0.066, *p* = 0.948 NS; *t* = 0.192, *p* = 0.848 NS.

**Table 8:** Distribution of total numbers of patients according to type of angulations of impaction. Data presented as frequency and percentage.

Angulations of impaction	Bilateral impaction		Unilateral impaction	
	Frequency	Percentage	Frequency	Percentage
Vertical	523	39.11	330	38.82
Mesioangular	549	41.07	333	39.18
Horizontal	51	3.82	33	3.88
Distoangular	209	15.63	149	17.53
Others	5	0.37	5	0.59
Total	1337	100.0	850	100.0

*χ*^2^ = 2.140, *p* = 0.710 NS.

**Table 9:** Distribution of total number of patients according to type of angulations of impaction in unilateral impaction. Data presented as frequency and percentage.

Angulations of impaction	Tooth number 38		Tooth number 48	
	Frequency	Percentage	Frequency	Percentage
Vertical	160	38.46	170	39.17
Mesioangular	157	37.74	176	40.55
Horizontal	18	4.33	15	3.46
Distoangular	79	19.0	70	16.13
Others	2	0.47	3	0.69
Total	416	100.0	434	100.0

*χ*^2^ = 2.023, *p* = 0.731 NS.

**Table 10:** Distribution of total number of patients according to level of impaction. Data presented as frequency and percentage.

Level of impaction	Bilateral impaction		Unilateral impaction	
	Frequency	Percentage	Frequency	Percentage
Level A	642	48.02	459	54.00
Level B	441	32.98	230	27.05
Level C	254	19.0	161	18.95
Total	1337	100.0	850	100.0

*χ*^2^ = 9.641, *p* = 0.008 S.

**Table 11:** Distribution of total number of patients according to level of angulations of impaction in unilateral impaction. Data presented as frequency and percentage.

Level of impaction	Tooth number 38		Tooth number 48	
	Frequency	Percentage	Frequency	Percentage
Level A	225	54.08	232	53.45
Level B	112	26.93	118	27.19
Level C	79	18.99	84	19.36
Total	416	100.0	434	100.0

*χ*^2^ = 0.036, *p* = 0.982 NS.

**Table 12:** Interexaminer reliability result based on angulation of impaction and level of impaction.

Cronbach's alpha	Cronbach's alpha
Based on angulations	0.991
Based on level of impaction	0.947
Based on angulations (2 weeks)	0.997
Based on level of impaction (2 weeks)	0.960

**Table 13:** Intraexaminer reliability result based on angulation of impaction.

Cronbach's alpha	Based on angulations
Examiner 1	0.979
Examiner 2	1.000
Examiner 3	0.998
Examiner 4	1.000

**Table 14:** Intraexaminer reliability result based on level of impaction.

Cronbach's alpha	Based on level of impaction
Examiner 1	0.960
Examiner 2	0.989
Examiner 3	0.990
Examiner 4	1.000

## Data Availability

All data are available within the manuscript.

## References

[1] Nazir A., Akhtar M. U., Ali S. (2014). Assessment of different patterns of impacted mandibular third molars and their associated pathologies. *Journal of Advanced Medical and Dental Sciences Research*.

[2] Hatem M., Bugaighis I., Taher E. M. (2016). Pattern of third molar impaction in Libyan population: a retrospective radiographic study. *The Saudi Journal for Dental Research*.

[3] Anwar N., Khan A. R., Narayan K. A., Manan A. H. (2008). A six year review of the third molar cases treated in the dental department of Penang hospital in Malaysia. *Dental Research Journal*.

[4] Khawaja N. A. (2006). Third molar impaction: a review. *Journal of the Pakistan Dental Association*.

[5] Reddy K. V. G. (2012). Distribution of third molar impactions among rural and urban dwellers in the age group of 22–30 years in South India: a comparative study. *Journal of Maxillofacial and Oral Surgery*.

[6] Haq Z. (2002). A survey of reasons for surgical removal of impacted mandibular third molar in armed forces personnel at AFID Rawalpindi. *Pakistan Oral & Dental Journal*.

[7] Wahid A., Mian F. I., Bokhan S. A., Moazzam A., Kramat A., Khan F. (2013). Prevalence of impacted mandibular and maxillary third molars: a radiographic study in patients reporting Madina teaching hospital, Faisal abad. *JUMDC*.

[8] Sujon M. K., Alam M. K., Enezei H. H., Rahman S. A. (2015). Third molar impaction and agenesis: a review. *International Journal of Pharma and Bio Sciences*.

[9] Olasoji H. O., Odusanya S. A. (2000). Comparative study of third molar impaction in rural and urban areas of South-Western Nigeria. *Odonto-Stomatologie Tropicale*.

[10] Ahlqwist M., Grondahl H.-G. (1991). Prevalence of impacted teeth and associated pathology in middle-aged and older Swedish women. *Community Dentistry and Oral Epidemiology*.

[11] Winter G. B. (1926). *The Principles of Exodontia as Applied to the Impacted Third Molar*.

[12] Pell G. J., Gregory B. T. (1933). Impacted mandibular third molars: classification and modified techniques for removal. *Dental Digest*.

[13] Svendsen H., JKM M., Andreasen J. O., Petersen J. K., Laskin D. M. (1997). Etiology of third molar impaction. *Textbook and Color Atlas of Tooth Impactions*.

[14] Alfergani S. M., Latif K., Alanazi Y. M. (2017). Pattern of impacted mandibular third molars in a Saudi population. *Pakistan Oral & Dental Journal*.

[15] Hassan A. H. (2010). Pattern of third molar impaction in a Saudi population. *Clinical, Cosmetic and Investigational Dentistry*.

[16] Syed K. B., Zaheer K. B., Ibrahim M., Bagi M. A., Assiri M. A. (2013). Prevalence of impacted molar teeth among Saudi population in Asir region, Saudi Arabia – a retrospective study of 3 years. *Journal of International Oral Health*.

[17] Haidar Z., Shalhoub S. Y. (1986). The incidence of impacted wisdom teeth in a Saudi community. *International Journal of Oral and Maxillofacial Surgery*.

[18] Alfadil L., Almajed E. (2020). Prevalence of impacted third molars and the reason for extraction in Saudi Arabia. *The Saudi Dental Journal*.

[19] Othman R. (2009). Impacted mandibular third molars among patients attending Hospital Universiti Sains Malaysia. *Archives of Orofacial Sciences*.

[20] Saiar M., Rebellato J. (2004). Maxillary impacted canine with congenitally absent premolars. *The Angle Orthodontist*.

[21] Byahatti S., Ingafou M. S. H. (2012). Prevalence of eruption status of third molars in Libyan students. *Dental Research Journal*.

[22] Maglutac M., Sarmiento M. A., Echiverre N. (2008). Impacted maxillary premolar: a report of two cases. *Emilio Aguinaldo College Research Bulletin*.

[23] Quek S. L., Tay C. K., Tay K. H., Toh S. L., Lim K. C. (2003). Pattern of third molar impaction in a Singapore Chinese population: a retrospective radiographic survey. *International Journal of Oral and Maxillofacial Surgery*.

[24] Niedzielska I. A., Drugacz J., Kus´ N., Kręska J. (2006). Panoramic radiographic predictors of mandibular third molar eruption. *Oral Surgery, Oral Medicine, Oral Pathology, Oral Radiology, and Endodontics*.

[25] Kaya G. S., Aslan M., Omezli M. M., Dayi E. (2010). Some morphological features related to mandibular third molar impaction. *Journal of Clinical and Experimental Dentistry*.

[26] Hugoson A., Kugelberg C. F. (1988). The prevalence of third molars in a Swedish population. An epidemiological study. *Community Dental Health*.

[27] Murtomaa H., Turtola I., Ylipaavalniemi P., Rytömaa I. (1985). Status of the third molars in the 20- to 21-year-old Finnish university population. *Journal of American College Health*.

[28] Rajasuo A., Murtomaa H., Meurman J. H. (1993). Comparison of the clinical status of third molars in young men in 1949 and in 1990. *Oral Surgery, Oral Medicine, and Oral Pathology*.

[29] Šečić S., Prohić S., Komšić S., Vuković A. (2013). Incidence of impacted mandibular third molars in population of Bosnia and Herzegovina: a retrospective radiographic study. *Journal of Health Sciences*.

[30] Hattab F. N., Fahmy M. S., Rawashedeh M. A. (1995). Impaction status of third molars in Jordanian students. *Oral Surgery, Oral Medicine, Oral Pathology, Oral Radiology, and Endodontology*.

[31] Eliasson S., Heimdahl A., Nordenram A. (1989). Pathological changes related to long-term impaction of third molars: A radiographic study. *International Journal of Oral and Maxillofacial Surgery*.

[32] Nejat A., Shamsabadi R. M., Rezaei N. M., Eshghpour M., Nezadi A., Moradi A. (2014). Pattern of mandibular third molar impaction: a cross-sectional study in northeast of Iran. *Nigerian Journal of Clinical Practice*.

[33] Shujaat S., Abouelkheir H. M., al-Khalifa K. S., al-Jandan B., Marei H. F. (2014). Pre-operative assessment of relationship between inferior dental nerve canal and mandibular impacted third molar in Saudi population. *The Saudi Dental Journal*.

[34] Gupta S., Bhowate R. R., Nigam N., Saxena S. (2011). Evaluation of impacted mandibular third molars by panoramic radiography. *ISRN Dentistry*.

[35] Dachi S. F., Howell F. V. (1961). A survey of 3,874 routine full-mouth radiographs: I. A study of impacted teeth. *Oral Surgery, Oral Medicine, Oral Pathology*.

[36] Padhye M. N., Dabir A. V., Girotra C. S., Pandhi V. H. (2013). Pattern of mandibular third molar impaction in the Indian population: a retrospective clinico-radiographic survey. *Oral Surgery, Oral Medicine, Oral Pathology and Oral Radiology*.

[37] Kramer R. M., Williams A. C. (1970). The incidence of impacted teeth: A survey at Harlem Hospital. *Oral Surgery, Oral Medicine, Oral Pathology*.

[38] Hazza's A. M., Albashaireh Z. S., Bataineh A. (2006). The relationship of the inferior dental canal to the roots of impacted mandibular third molars in Jordanian population. *J Contemp Dent Pract*.

[39] Hellman M. (1936). Our third molar teeth: their eruption, presence and absence. *Dent Cosmos*.

[40] Scherstén E., Lysell L., Rohlin M. (1989). Prevalence of impacted third molars in dental students. *Swedish Dental Journal*.

[41] Ma’aita J., Alwrikat A. (2000). Is the mandibular third molar a risk factor for mandibular angle fracture?. *Oral Surgery, Oral Medicine, Oral Pathology, Oral Radiology, and Endodontology*.

[42] Almendros-Marqués N., Berini-Aytés L., Gay-Escoda C. (2006). Influence of lower third molar position on the incidence of preoperative complications. *Oral Surgery, Oral Medicine, Oral Pathology, Oral Radiology, and Endodontology*.

[43] Sandhu S., Kaur T. (2005). Radiographic evaluation of the status of third molars in the Asian-Indian students. *Journal of Oral and Maxillofacial Surgery*.

